# Targeting hepatocyte growth factor in epithelial–stromal interactions in an in vitro experimental model of human periodontitis

**DOI:** 10.1007/s10266-021-00625-0

**Published:** 2021-06-14

**Authors:** Yoko Yamaguchi, Akira Saito, Masafumi Horie, Akira Aoki, Patrick Micke, Mitsuhiro Ohshima, Kai Kappert

**Affiliations:** 1 Department of Biochemistry, Nihon University School of Dentistry, Tokyo, Japan; 2 Division of Functional Morphology, Dental Research Center, Nihon University School of Dentistry, Tokyo, Japan; 3 Department of Respiratory Medicine, Graduate School of Medicine, The University of Tokyo, Tokyo, Japan; 4 Department of Cancer Genome Informatics, Graduate School of Medicine, Osaka University, Osaka, Japan; 5 Department of Periodontology, Graduate School of Medical and Dental Sciences, Tokyo Medical and Dental University, Tokyo, Japan; 6 Department of Immunology, Genetics and Pathology, Uppsala University, 751 85 Uppsala, Sweden; 7 Department of Biochemistry, Ohu University School of Pharmaceutical Sciences, Koriyama, Fukushima Japan; 8 Charité - Universitätsmedizin Berlin, Freie Universität Berlin, Humboldt-Universität zu Berlin, Institute of Laboratory Medicine, Clinical Chemistry and Pathobiochemistry, Augustenburger Platz 1, 13353 Berlin, Germany

**Keywords:** Hepatocyte growth factor, Periodontitis, Human primary cell, Extracellular matrix, Transcriptome

## Abstract

Periodontitis is a chronic inflammatory disease leading to progressive connective tissue degradation and loss of the tooth-supporting bone. Clinical and experimental studies suggest that hepatocyte growth factor (HGF) is involved in the dysregulated fibroblast–epithelial cell interactions in periodontitis. The aim of this study was to explore effects of HGF to impact fibroblast-induced collagen degradation. A patient-derived experimental cell culture model of periodontitis was applied. Primary human epithelial cells and fibroblasts isolated from periodontitis-affected gingiva were co-cultured in a three-dimensional collagen gel. The effects of HGF neutralizing antibody on collagen gel degradation were tested and transcriptome analyses were performed. HGF neutralizing antibody attenuated collagen degradation and elicited expression changes of genes related to extracellular matrix (ECM) and cell adhesion, indicating that HGF signaling inhibition leads to extensive impact on cell–cell and cell–ECM interactions. Our study highlights a potential role of HGF in periodontitis. Antagonizing HGF signaling by a neutralizing antibody may represent a novel approach for periodontitis treatment.

**Supplementary Information:**

The online version contains supplementary material available at 10.1007/s10266-021-00625-0.

## Introduction

Periodontitis is a chronic inflammatory process associated with loss of the tooth-supporting tissue [1, 2]. Dysbiosis of the oral microbiome [3–6] and host responses influenced by many factors are involved in the pathogenesis of periodontitis [7–11]. Further, presence of systemic diseases [12, 13] and gene polymorphisms [14–16] are reportedly correlated with the risk for periodontitis. Degradation of extracellular matrix (ECM) within the gingival connective tissue located between tooth and alveolar bone is the driving force in the pathological process of periodontitis [9, 17–20].

Activated fibroblasts are known to produce ECM components as well as proteolytic enzymes, thereby contributing to ECM remodeling and degradation [18, 21, 22]. Previously, we have isolated periodontitis-associated fibroblasts (PAFs) from the gingiva of periodontitis affected patients. These PAFs demonstrated a higher capacity of collagen degradation compared to normal gingival fibroblast and were characterized by a distinct gene expression profile [8, 9, 18]. To develop an experimental model of periodontitis, primary cultured gingival epithelial cells and PAFs were co-cultured in collagen gels, which appears to recapitulate epithelial cell–fibroblast interactions in the gingival connective tissue. In a series of previous reports, we have demonstrated that PAFs display a remarkably higher capacity of ECM degradation compared to normal fibroblasts [9, 18].

A number of cytokines and growth factors have been reported to participate in the pathological process of periodontitis. Previous studies demonstrated that hepatocyte growth factor (HGF) is secreted by gingival fibroblasts and is abundant in gingival crevicular fluid (GCF) of periodontitis patients, indicating its clinical relevance [23, 24]. Of importance, HGF levels were found to correlate with established predictors of periodontitis such as probing depth (PD), gingival index (GI), bleeding on probing (BOP), and bone resorption [24–27].

HGF represents a paracrine growth factor capable of enhancing cell motility and survival [28], and is implicated in the wound healing process [29]. Its cognate receptor, MET, is expressed in epithelial cells but not in mesenchymal cells or fibroblasts. HGF is significantly higher in both GCF and saliva from periodontitis patients compared to healthy individuals [23], potentially correlating with disease severity. Furthermore, Nagaraja et al. demonstrated that HGF levels in GCF decline after non-surgical periodontal therapy [24]. Thus, HGF levels in GCF might serve as both a companion diagnostic tool and a surrogate marker for disease progression or therapeutic response. Interestingly, upregulation of HGF is also known in cancer-associated fibroblasts that demonstrate tumorigenic properties [30, 31]. Given that PAFs constitute a major source of HGF in periodontitis, HGF may represent a hallmark of pathologically activated or disease-associated fibroblasts.

In our previous report, we identified 22 genes upregulated in PAFs-containing collagen gels through comprehensive gene expression profiling [8, 9, 18]. Among them, HGF showed clearly higher expression that was validated by quantitative PCR analysis [8, 9, 18]. Here, we sought to address whether HGF might serve as a therapeutic target utilizing a state-of-the art in vitro co-culture system as an experimental model of periodontitis. In this unique model, we use primary human gingival epithelial cells and primary fibroblasts from periodontitis affected patients. We show that targeting HGF by a neutralizing antibody alleviates collagen gel degradation. Our data might pave the way for HGF antagonism as a novel therapeutic approach against periodontitis.

## Materials and methods

### Cell culture

Gingival epithelial cells and fibroblasts were isolated from gingival tissues of periodontitis patients as described previously [8]. Briefly, excised gingival tissue was cut into small pieces and placed into 6-well plates. Gingival fibroblast populations were established from each well, which was pooled thereafter to one population. Cells were maintained in α-minimum essential medium (α-MEM, Wako, Osaka, Japan) supplemented with 10% fetal bovine serum (FBS, Hyclone, Logan, UT, USA) and 1% penicillin/streptomycin/neomycin. Gingival epithelial cells were maintained in EpiLife^®^ medium with calcium with S7 supplement (Thermo Fisher Scientific, Waltham, MA, USA), and grown in type I collagen-coated flasks (Sumitomo Bakelite, Tokyo, Japan). For the subsequent experiments cell cultures between the 7th and 15th passages were used.

The information of patients and donors for the experiments are indicated in Supplementary Table 1. The obtained samples are not matched to the disease grade of periodontitis and came from different patients. Gingival tissues were obtained during periodontal surgery at Nihon University School of Dentistry, Dental Hospital, and at the Department of Periodontics, Dental Hospital of Tokyo Medical and Dental University (TMDU). The protocol was approved by the Ethics Committee of Ohu University, Nihon University School of Dentistry, and the Faculty of Dentistry, TMDU. All patients gave written informed consent. The work has been carried out in accordance with the Code of Ethics of the World Medical Association (Declaration of Helsinki).

### Collagen gel co-culture assay

Three-dimensional (3D) co-culture of gingival epithelial cells and PAFs as an experimental model of periodontitis was carried out as described previously [8, 9, 32]. Briefly, collagen gels were prepared by mixing 0.5 mL of fibroblast cell suspension (2.5 × 10^5^ cells) in FBS, 2.3 mL of type I collagen (Cellmatrix type I-A; Nitta Gelatin Inc., Osaka, Japan), 670 µL of 5 × DMEM, and 330 µL of reconstitution buffer, following the manufacturer’s recommendations. The mixed solution (3 mL) was placed into each well of a 6-well plate and allowed to gelatinize in an incubator at 37 °C. Subsequently, 2.5 × 10^5^ gingival epithelial cells resuspended in 2 mL of EpiLife^®^ medium with Supplement S7 were seeded onto the surface of each gel. The gels were cultured overnight and separated from the edge of each well to generate a ‘floating culture’. The gels were then placed in the medium for 5 days and cultured for additional 5 days at the air–liquid interface. Gels were fixed in neutral buffered formalin solution and embedded in paraffin. Vertical sections were stained with hematoxylin and eosin. Collagen gels were treated with the indicated concentrations of recombinant HGF (#100-39, Peprotech, NJ, USA) or HGF neutralizing antibody (AB-294-NA, R&D Systems, Minneapolis, MN, USA). As the control for the polyclonal HGF neutralizing antibody, we used normal goat polyclonal IgG at the same concentration (AB-108-C, R&D Systems). The culture experiments were performed under the assumption that the final size of cultured gels reflect the degree of collagen degradation.

### Collagen assay

Gels were weighed and placed into microtubes, and distilled water was added to a total weight of 1.0 g. Thereafter, the tubes were heated at 80 °C for 1 h to dissolve the collagen. Each supernatant was used to measure collagen contents by Sircol™ Soluble Collagen Assay (Biocolor, Carrickfergus, County Antrim, UK), and the total amount of remaining collagen in the gel was calculated according to the manufacturer’s protocol [8, 9].

### Microarray analysis

Total RNA from the collagen gels after treatment with the HGF neutralizing antibody or the control goat IgG was isolated using RNeasy Mini Kit (Qiagen, Hilden, Germany). RNA samples were routinely monitored for RNA integrity on Bioanalyzer 2100 (Agilent Technologies, Wilmington, DE, USA). Gene expression profiles were analyzed using microarray technique (Affymetrix GeneChip™, Human Genome U133 Plus 2.0 Array, Santa Clara, CA, USA).

Genes that showed fold change of normalized values ≤ 0.67 or ≥ 1.5 in three independent cell culture experiments were used for comparative analyses.

### Quantitative real-time PCR

Quantitative real-time PCR was carried out for validating gene expression profiles detected in microarray analyses. Total RNA was extracted using the Trizol reagent (Invitrogen, Thermo Fisher Scientific, Waltham, MA, USA). The cDNA was synthesized using PrimerScript RT reagent kit (Takara, Shiga, Japan) following the manufacturer’s protocol. Quantification of mRNA levels was performed using SYBR Green (SYBR Premix Ex Taq II, Takara), and a PCR thermal cycler (TP900, Takara). Relative mRNA expression was calculated using the ΔΔCt method. The quantitative expression was normalized to the transcript levels of glyceraldehyde 3-phosphate dehydrogenase (GAPDH). The primer pairs used are outlined in Supplementary Table 2.

### Descriptive statistical analysis

Student’s *t* test for paired samples was performed. *P* values < 0.05 were considered as statistically significant.

## Results

An in vitro experimental model of human periodontitis was applied, where gingival epithelial cells and PAFs were co-cultured in collagen gels (Supplementary Fig. 1A). Using this model, we have previously demonstrated that PAFs are highly capable of collagen degradation in clear contrast to control fibroblasts [8, 9, 18]. The cross section of the collagen gel after the culture period of total 10 days illustrates the changes in collagen gel shape and cellular components in the superficial epithelial layer (Supplementary Fig. 1B).

Previously, we have also provided primary cultured gingival epithelial cells and fibroblasts for the FANTOM5 project [33], which enabled us to identify transcription start sites across the whole genome using the Cap Analysis of Gene Expression sequencing technology [34]. We utilized this dataset and compared expression levels of HGF and its cognate receptor, MET, in these cell types. The analysis revealed MET being predominantly expressed in gingival epithelial cells, whereas HGF appeared to be exclusively expressed in gingival fibroblasts (Table 1).

**Table 1:** CAGE tag counts of transcription start sites (p1 promoters) annotated to HGF and MET (average ± SD)

	HGF	MET
Gingival fibroblasts (*n* = 6)	91.1 ± 46.4	21.8 ± 38.0
Gingival epithelial cells (*n* = 3)	0 ± 0	119.1 ± 39.6

CAGE data were compared between gingival epithelial cells and fibroblasts

**Fig. 1:**
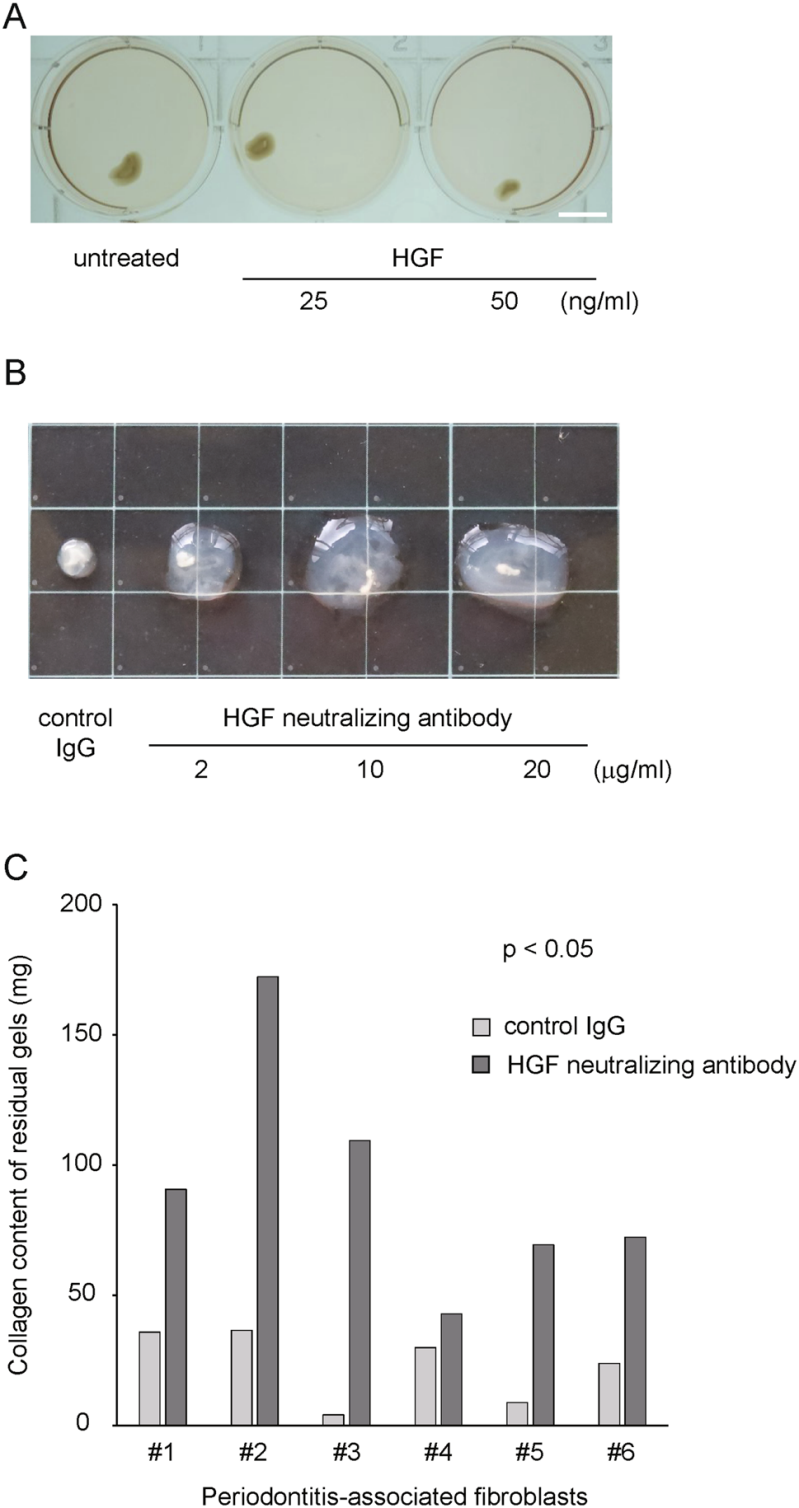
Effects of recombinant HGF or HGF neutralizing antibody on collagen gel degradation. **a** Effect of HGF (25 or 50 ng/mL) on collagen gel degradation. Representative gels are shown. Scale bar: 1 mm. **b** Effect of HGF neutralizing antibody on collagen gel degradation. Three-dimensional co-culture gels were treated with control IgG (normal goat 20 µg/mL) or different concentrations of HGF neutralizing antibody (2, 10 and 20 µg/mL). Representative gels are shown. **c** Three-dimensional co-culture gels containing gingival epithelial cells and PAFs were assessed with regard to collagen gel degradation. The residual collagen gel content was quantified (*n* = 6). HGF neutralizing antibody was used at the concentration of 10 µg/mL

We hypothesized that HGF signaling-mediated cellular interactions might impact on periodontitis progression. First, gingival epithelial cells and PAFs were co-cultured in collagen gels and treated with recombinant HGF. Exogenous HGF did not have a substantial impact on collagen gel degradation (Fig. 1A). Next, gingival epithelial cells and PAFs co-cultured in collagen gels were treated with different concentrations of HGF neutralizing antibody and collagen gel degradation was monitored. The HGF neutralizing antibody attenuated collagen gel degradation in a concentration-dependent manner (Fig. 1B). The residual collagen gel content was significantly higher under the HGF neutralizing antibody treatment (Fig. 1C).

**Fig. 2:**
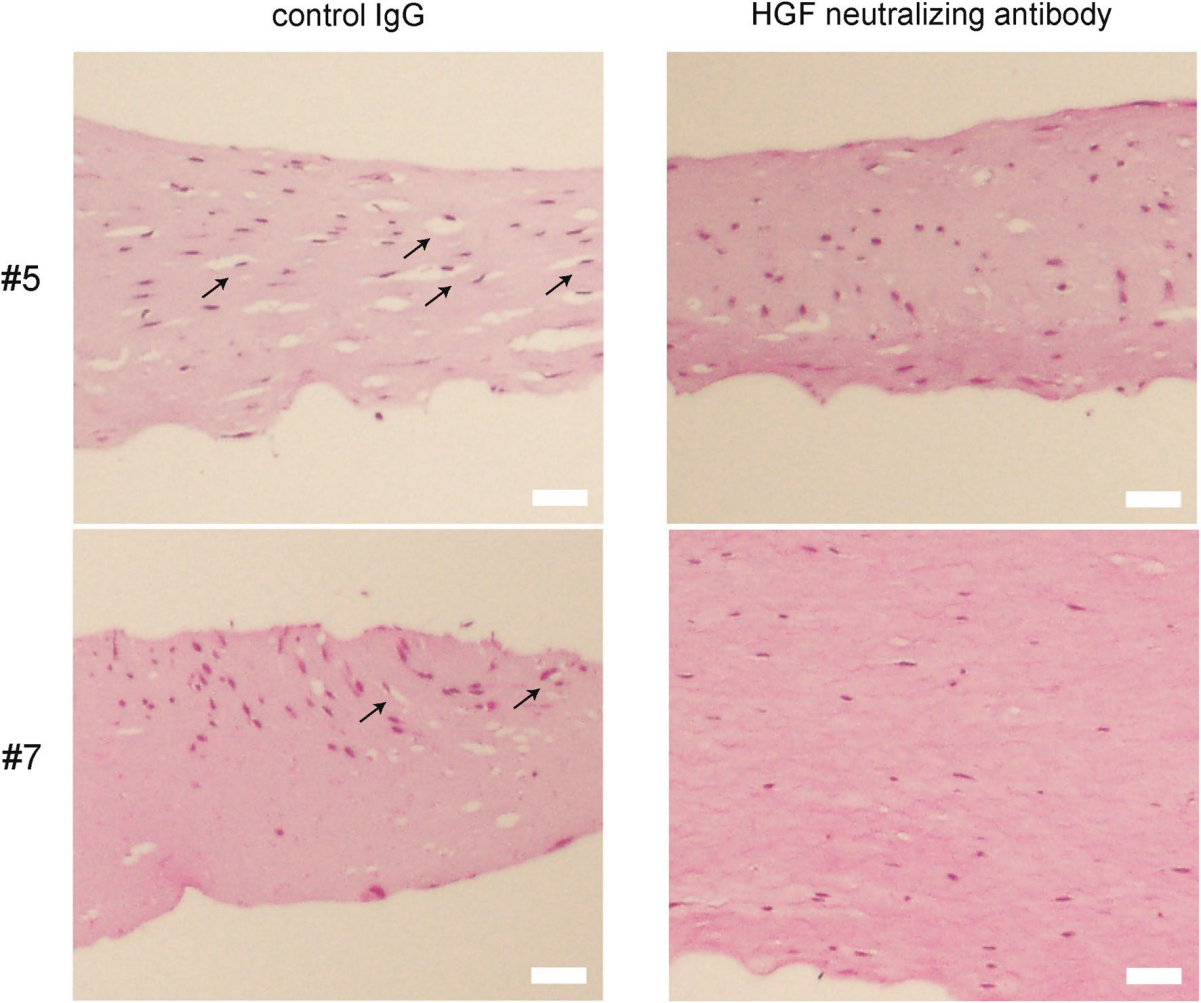
Impact of HGF neutralizing antibody on vacuolization in collagen gels. Three-dimensional co-culture gels containing PAFs derived from periodontitis patient #5 and #7 were treated with control IgG or HGF neutralizing antibody (10 µg/mL). Collagen gels were paraffin-embedded and sections were stained with hematoxylin–eosin. Treatment with HGF neutralizing antibody resulted in a reduction of vacuole numbers. Arrows point to vacuoles surrounding PAFs observed in the control gel. The numbers of vacuoles decreased under the HGF neutralizing antibody treatment (10 µg/mL). Scale bar: 50 μm

Further histological observations of 3D collagen gel cultures revealed reduced number of vacuoles surrounding fibroblasts under the HGF neutralizing antibody treatment (Fig. 2).

The percentages of fibroblasts associated with apparent vacuoles were 24.2 ± 8.2% (average ± SD) in the control group and 8.8 ± 6.2% in the HGF neutralizing antibody group.

**Fig. 3:**
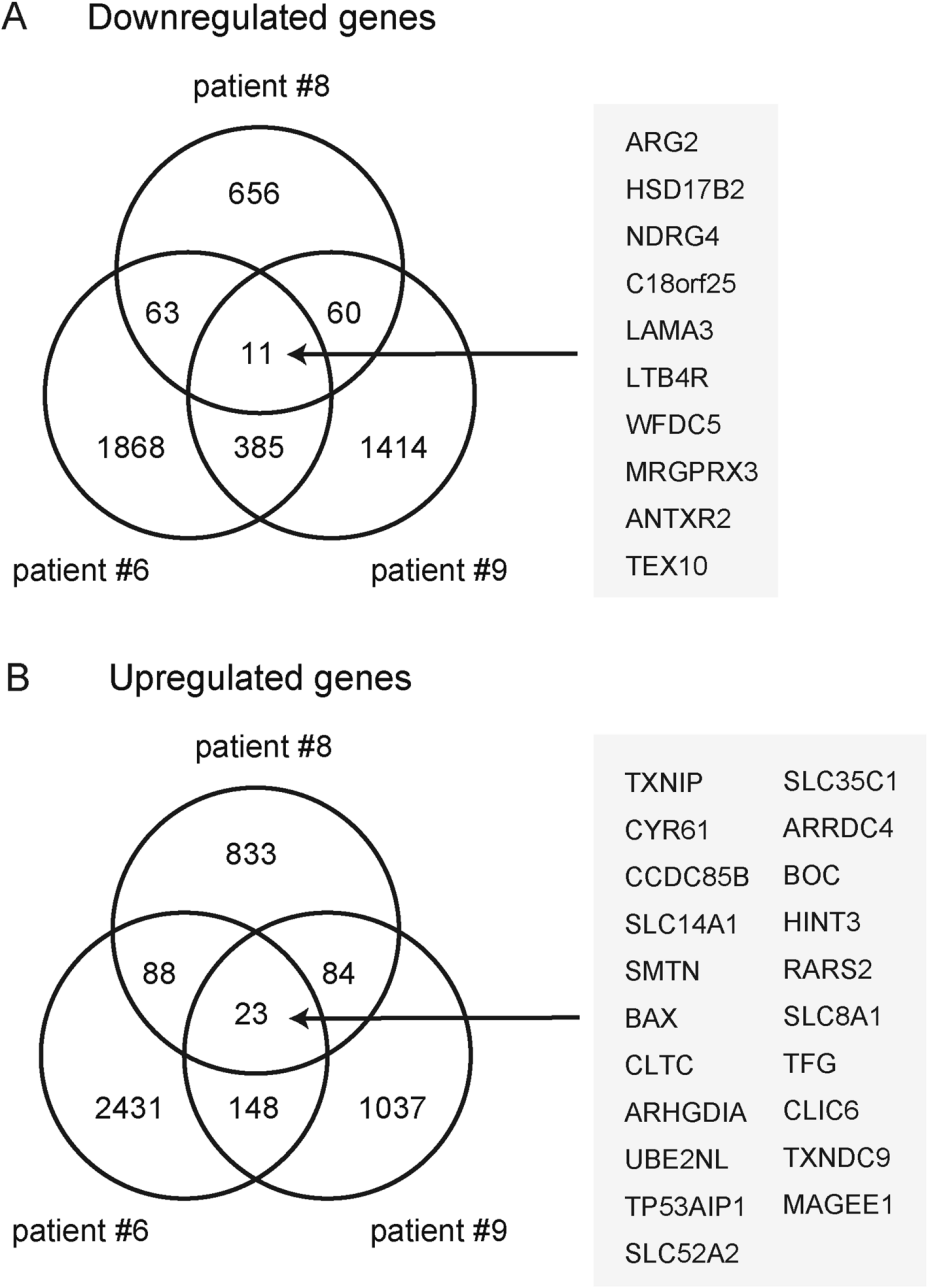
Gene expression profiling of collagen gels after anti-HGF treatment. RNA was extracted from collagen gel co-cultures of PAFs and gingival epithelial cells treated with HGF neutralizing antibody treatment (10 µg/mL) or control IgG. Venn diagram illustrates the **a** downregulated or **b** upregulated transcripts in three independent collagen gels containing PAFs derived from three individuals (periodontitis patient #6, #8, and #9). Numbers of transcripts are indicated. Annotated genes that were concordant in three independent experiments are highlighted

Finally, we performed gene expression profiling of 3D collagen gel cultures that contained gingival epithelial cells and PAFs in the presence or absence of the HGF neutralizing antibody. Three preparations of independently isolated co-cultures were monitored for concordant gene upregulation or downregulation. Using thresholds of fold change of normalized values ≤ 0.67 or ≥ 1.5, we identified 11 gene that were downregulated and 23 transcripts that were upregulated in response to anti-HGF treatment (Fig. 3A, B). These transcripts corresponded to 10 and 21 annotated genes, respectively, and the summary of these genes and general descriptions of their specific functions are depicted in Supplementary Table 3A and 3B.

**Fig. 4:**
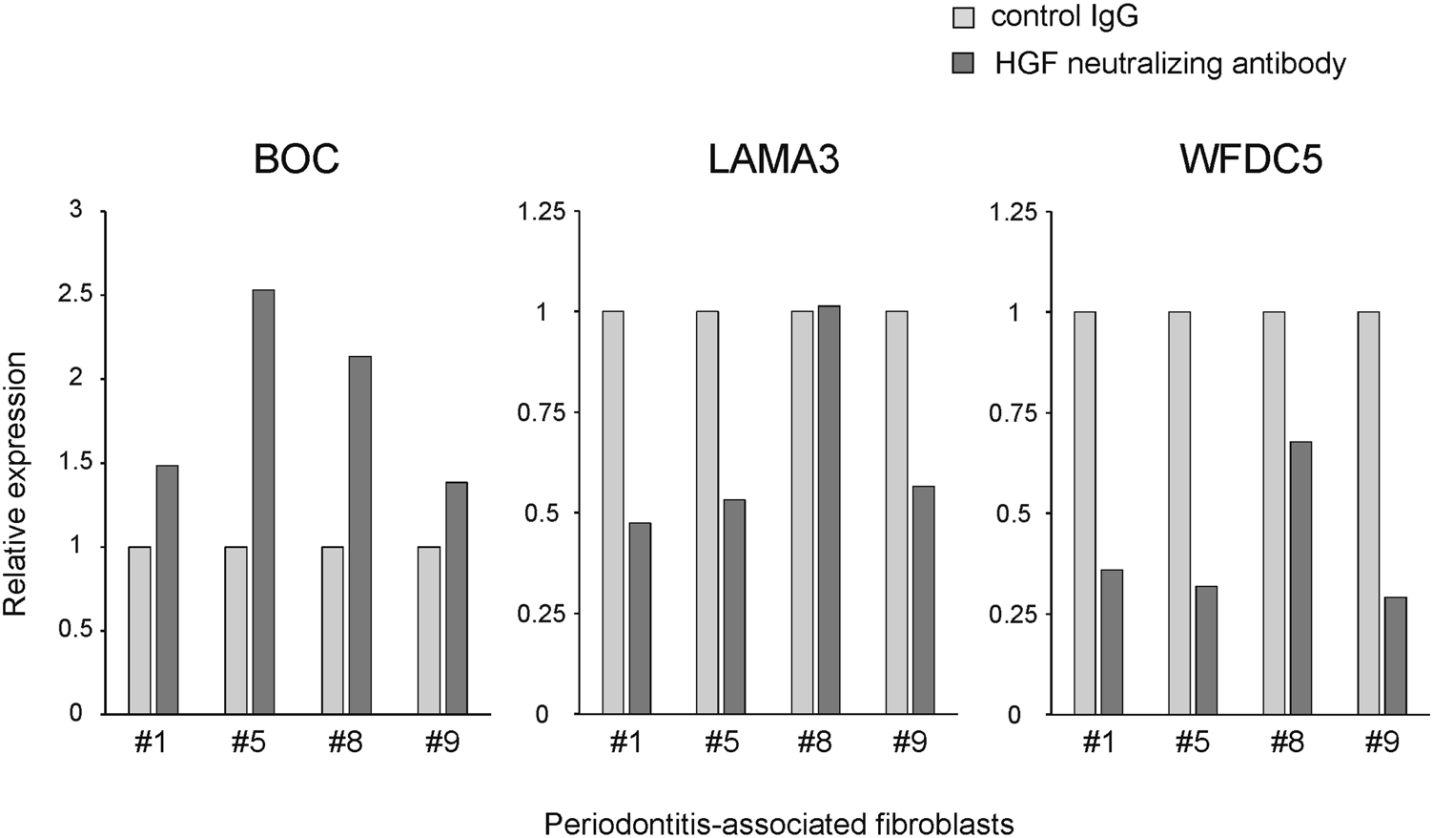
Gene expression changes after anti-HGF treatment for selected genes by quantitative real-time PCR. Gene expression changes in four different co-cultures models treated with HGF neutralizing antibody (10 µg/mL) or control IgG. Collagen gels-containing PAFs derived from four individuals (periodontitis patient #1, #5, #8 and #9) were evaluated. The mRNA levels of BOC (BOC Cell Adhesion Associated, Oncogene Regulated), LAMA3 (Laminin Subunit Alpha 3) and WFDC5 (WAP Four-Disulfide Core Domain 5) were normalized to the expression of the house-keeping gene GAPDH. PAFs #8 and #9 were also used for microarray analysis

We confirmed the expression changes indicated by the microarray experiments by real-time PCR for the genes BOC, LAMA3 and WFDC5 (Fig. 4).

Taken together with our observation that antagonizing HGF results in alleviated collagen degradation in 3D co-cultures, these data implicated that HGF signaling drives expression changes in a subset of genes that are involved in ECM turnover underlying the pathogenesis of periodontitis.

## Discussion

Effective treatment of periodontitis represents a major clinical challenge. This is at least partially due to the fact that the molecular and cellular pathogenesis of periodontitis is only fragmentarily understood. In our previous studies, we have demonstrated that PAFs constitute a key component in the pathogenesis of periodontitis. This is based on the observations that PAFs are highly capable of matrix degradation in an in vitro experimental model of periodontitis, which is absent when normal fibroblasts are used [8, 9, 18]. Thus, targeting differentially regulated soluble factors that are associated with tissue destruction can be a novel therapeutic strategy against periodontitis. Here, we show that antagonizing HGF via an antibody-based approach resulted in reduced collagen degradation of 3D co-cultures, suggesting that HGF pathway inhibition contains valuable therapeutic potential.

Extensive experimental and clinical data provide several lines of evidence that HGF and its cognate receptor MET are crucial components of various diseases including organ fibrosis and tumor progression [35]. In periodontitis, a positive association has been described between concentrations of HGF in saliva and alveolar bone loss [36]. We and others further showed that HGF concentration is upregulated in GCF of periodontitis patients [24, 26, 27, 37]. We also demonstrated that PAFs are characterized by enhanced HGF gene expression [8, 9, 18], suggesting PAFs as a major source of HGF production in periodontitis.

Here, we further addressed HGF as a potential molecular target in an experimental model of periodontitis using a unique human primary cells and 3D co-culture system. In contrast to many other experimental in vitro models [38, 39], we utilize both, epithelial and mesenchymal primary cells isolated from patient tissue. As a functional and objective read-out, collagen gel degradation was assessed. The finding that PAFs led to the typical collagen degradation, in contrast to normal fibroblasts, indicated that our 3D co-culture system seemed to recapitulate cellular interactions between the epithelium and fibroblasts in the gingival tissue of periodontitis.

An antibody-based anti-HGF approach resulted in an inhibition of collagen gel degradation in this in vitro model, suggesting HGF signaling being crucially involved in ECM turnover, and antagonism of HGF representing a valid treatment approach to ameliorate periodontitis. Furthermore, vacuoles in 3D co-cultures were reduced upon HGF neutralizing antibody treatment, indicating a reduction of para-cellular matrix degradation. Given the low expression of MET in fibroblasts, the effect of HGF blocking is likely mediated by epithelial cells that participate in fibroblast activation. One of these factors can be TGF-β that is implicated in stroma remodeling in inflammation, wound healing and tumorigenesis. Previously we demonstrated that TGF-β type I receptor kinase inhibitor (ALK5 inhibitor) effectively inhibits collagen gel degradation [8], and a similar reduction of perifibroblast vacuolization was observed in this study. Thus, it is possible that the TGF-β pathway is also functionally involved in the development of periodontitis and is connected to HGF signaling.

We further addressed gene expression changes in 3D co-cultures after HGF neutralizing antibody treatment. A variety of genes was significantly upregulated or downregulated upon treatment. Gene Ontology analysis did not reveal any significant biological motives, most likely due to the small number of genes and rigorous adjustment for multiple testing.

However, some of the genes may deserve attention since they may mediate important effects of anti-HGF treatment. Among the upregulated genes, we identified CYR61/CCN1, interacting with several integrins and thus cell–ECM interactions [40], BAX, involved in apoptosis regulation [41], and BOC, a member of the immunoglobulin/fibronectin type III repeat family and thus impacting on cell–cell interactions [42]. With regard to downregulated genes, we identified ANTXR2 and LAMA3, both being implicated in ECM interactions [43, 44]. Further, leukotriene B4 receptor was also downregulated, suggesting a potential modification of the inflammatory status [45, 46]. However, it needs to be stressed that the definite roles of the identified genes, being either down- or upregulated, remain to be determined with regard to their specific impact on periodontitis remodeling.

A limitation of our study lies in the fact that our experimental model reflects only a few aspects of the complex interaction of different cell types in periodontitis and presents therefore a relatively reductive approach. It is likely that several signal pathways are involved in the reciprocal interactions between gingival epithelial cells and fibroblasts, and HGF might represent only one of many important signals. Also not included in our model are inflammatory cells that give raise to heterogeneous context dependent signals. In addition, periodontitis has many clinical phenotypes, but we did not differentiate the disease status of periodontitis patients in this study. In addition, the tissue procurement may imply a bias, because at the time of operation patient hygiene is usually improved. Thus, phenotypic differences in relationship to the severity of periodontitis and the inflammation status would be informative in future studies. Furthermore, analyzing different time points following HGF signaling inhibition might reveal HGF-associated phenotypic and molecular switch of fibroblasts in more detail.

In summary, our data point towards the relevance of HGF in periodontitis-associated tissue remodeling and suggest the use of HGF neutralizing antibody treatment as a novel therapeutic approach.

## Supplementary Information

Below is the link to the electronic supplementary material.

Supplementary file1. Supplementary Figure 1 Overview of three-dimensional co-culture method as an in vitro experimental model of periodontitis. **A** Schematic representation of three-dimensional co-culture of gingival epithelial cells and periodontitis-associated fibroblasts (PAFs). Collagen gels were cultured at the air-liquid interface. **B** Cross section of the collagen gel after the culture period of total 10 days. Scale bar: 100 µm. (PDF 1480 kb)

Supplementary file2 (XLS 66 kb)
