# Supplementary figures and images for: Targeting hepatocyte growth factor in epithelial–stromal interactions in an in vitro experimental model of human periodontitis
Source: Odontology. 2021 Jun 14;109(4):912–20. doi: 10.1007/s10266-021-00625-0 (PMC8387255; doi:10.1007/s10266-021-00625-0)

A

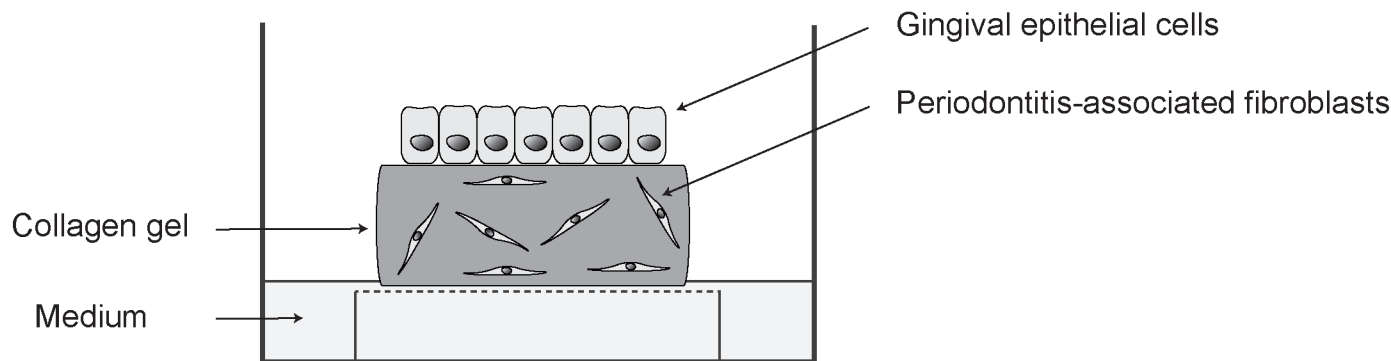

B

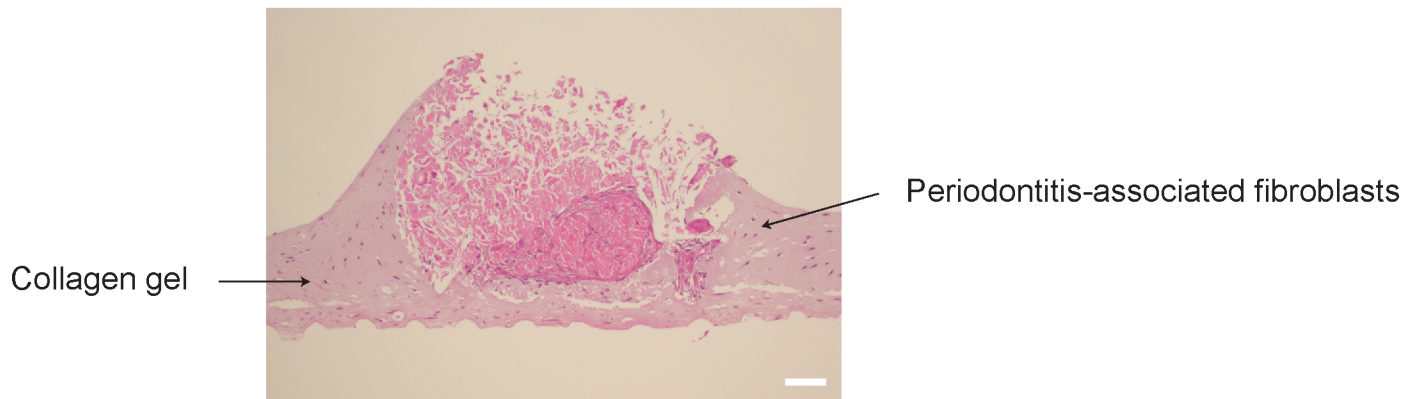

Supplement: Supplementary file 1 — Supplementary file1. Supplementary Figure 1 Overview of three-dimensional co-culture method as an in vitro experimental model of periodontitis. A Schematic representation of three-dimensional co-culture of gingival epithelial cells and periodontitis-associated fibroblasts (PAFs). Collagen gels were cultured at the air-liquid interface. B Cross section of the collagen gel after the culture period of total 10 days. Scale bar: 100 µm. (PDF 1480 kb) [file 10266_2021_625_MOESM1_ESM.pdf]
